# Response to the letter by de Boer et al. (2022)

**DOI:** 10.1186/s40478-022-01474-w

**Published:** 2022-11-29

**Authors:** Karri Kaivola, Pentti J. Tienari

**Affiliations:** 1 Translational Immunology Research Program, University of Helsinki, Helsinki, Finland; 2 Neurocenter, Helsinki University Hospital, Helsinki, Finland

The hexanucleotide repeat expansion (HRE) in the *C9orf72* gene is the most common genetic cause of amyotrophic lateral sclerosis (ALS) and frontotemporal dementia (FTD) in European ancestry populations and especially common in Finland [1, 2]. Whether there are risk alleles or haplotypes in the *C9orf72* locus, besides the HRE, is an important question, because the putative non-expansion risk alleles are more common in the general population than the HRE.

We have previously reported that carriership of two copies of intermediate-length alleles (IAs) associate with ALS, when one of the alleles is ≥ 17 repeats [3]. De Boer et al. could not confirm this association in a large multi-center study [4]. We believe that our findings are essentially true, but the lack of confirmation may be caused by imprecisely assigned repeat allele threshold, haplotype heterogeneity, population stratification, or IA calling inconsistencies.

Van der Zee et al. [5] were the first to report that homozygosity for a single-nucleotide polymorphism (SNP) (rs2814707) was associated with FTD in a Flanders-Belgian case-control study after excluding expansion carriers [odds ratio (OR) 1.75, 95% CI 1.02-3.0, *p* = 0.04]. This SNP tags the HRE and IAs with ≥ 7 repeats. In Belgian ALS and FTD–ALS patients a significantly increased risk was found for carriers of two copies of the IAs (OR 2.08, 95%CI 1.04–4.1, *p* = 0.04), tagging SNPs were not reported [6].

We reported that homozygosity for the IAs (≥ 7/≥7 genotype, OR 1.93, 95%CI 1.19–3.02) and tagging SNP (A/A genotype rs3849942, OR 1.89, 95%CI 1.20–2.99) associated with ALS with similar magnitude in the Finnish population [3]. In our data we did not detect any significant ALS risk with IA genotypes 7–16/7–16 (OR 1.57, 95%CI 0.90–2.62), while an increasing risk of ALS was found with genotypes ≥ 7/≥17, ≥ 7/≥21 and ≥ 7/≥24. Thus, IA length influenced the magnitude of the risk and the threshold for significant risk was with the genotype ≥ 7/≥17. IA length also increased the correlation with the tagging SNP in our data [3] and in the UK birth cohort [7]. Thus, the haplotype becomes more homogeneous with increasing IA length.

Two of the previous studies reported association of ALS or FTD with tagging SNP homozygosity [3, 5] and two studies with intermediate length allele homozygosity [3, 6]. Now, the largest study to date did not confirm the reported risk conferred by IA homozygosity. What might be the explanation for these varying results?

First, the cutoff (≥ 17 repeats) for the IAs may be nonoptimal. De Boer et al. used this cutoff (genotype ≥ 7/≥17) for the analysis of longer IAs, the cutoffs ≥ 21 and ≥ 24 were not reported. We have found that the frequency of alleles of ≥ 20 repeats is 2-fold higher among Finns (0.89%) than in other populations [8], including UK 1958 Birth cohort (0.42%). However, according to published data [7, 8], the alleles with 17–19 repeats are more common the UK birth cohort (0.53%) than in the Finnish populations (0.30%). Thus, based on the geographic enrichment of longer IAs (≥ 20 repeats) in Finland and increasing haplotypic homogeneity associated with longer IAs, it is possible that these alleles/haplotypes are relevant for disease risk. There is also biological evidence from transfected cells (11, 20, 22 and 41 repeats tested) indicating that instability of the repeat is length-dependent and replication fork stalling is observed at 20 repeats [9]. In the future, a higher cutoff should be tested. Also, somatic instability and resulting mosaicism should be analyzed in ALS and FTD patients with ≥ 20 repeat alleles.

Second, as discussed by de Boer et al., haplotype heterogeneity is one possibility and further studies are needed to explore which part(s) of these haplotypes are playing a role in ALS/FTD risk. This risk may be conferred by the IAs as such, other variation on the haplotype or their combination. Haplotype heterogeneity regarding the best expansion-tagging SNPs has already been reported between Finnish and UK populations [10] and the above-mentioned differences in the frequencies of the 17–19 and ≥ 20 repeat alleles may reflect differences in the haplotype background of these alleles.

Third, all three studies that reported associations with ALS or FTDs were single-population studies [3, 5, 6]. In the study of de Boer et al. data was collected from six different publications with three cohorts from Netherlands, two from UK, two from Italy, one from Spain and one from North America. Of the controls 80% were derived from the UK 1958 birth cohort, while 82% of the ALS patients and 37% of the FTD patients (largest fraction) were from Netherlands. Risk haplotypes may be differentially distributed in these cohorts, which may affect the results; tagging SNPs were not available to control for haplotype heterogeneity. In the future single-population analyses of ALS/FTD with combined IA and SNP genotyping should be tested to replicate the reported findings. It will also be important to replicate the findings in Finnish FTD cases.

Fourth, genotyping methodology may influence the allele calling. In our study all *C9orf72* repeat allele lengths were assessed in the same laboratory contrary to what was mentioned in the de Boer et al. letter (rs3849942 genotyping was performed at multiple sites). We also made additional over-the-repeat PCRs to verify the larger IA lengths. In the cohorts reported by de Boer et al. slightly different RP-PCR methodologies were used and the largest ALS cohort that contributed 82% of the ALS cases was genotyped by short-read whole genome sequencing data and *in silico* with the ExpansionHunter algorithm. Sizing of the longer IAs is less accurate than expansion detection with short-read sequencing. In future studies aiming to replicate the previous findings a similar IA genotyping methodology should preferably be used.

These varying results should stimulate the field to define the *C9orf72* haplotypes more in detail (e.g. by the use of long-range sequencing) to uncover the putative expansion-independent genetic effect of this ALS/FTD locus.

## References

[1] Majounie E, Renton AE, Mok K, Dopper EG, Waite A, Rollinson S, Chio A, Restagno G, Nicolaou N, Simon-Sanchez J, van Swieten JC, Abramzon Y, Johnson JO, Sendtner M, Pamphlett R, Orrell RW, Mead S, Sidle KC, Houlden H, Rohrer JD, Morrison KE, Pall H, Talbot K, Ansorge O, Consortium ITALSGEN, Hernandez DG, Arepalli S, Sabatelli M, Mora G, Corbo M, Giannini F, Calvo A, Englund E, Borghero G, Floris GL, Remes AM, Laaksovirta H, McCluskey L, Trojanowski JQ, Van Deerlin VM, Schellenberg GD, Nalls MA, Drory VE, Lu CS, Yeh TH, Ishiura H, Takahashi Y, Tsuji S, Le Ber I, Brice A, Drepper C, Williams N, Kirby J, Shaw P, Hardy J, Tienari PJ, Heutink P, Morris HR, Pickering-Brown S, Traynor BJ (2012). Frequency of the C9orf72 hexanucleotide repeat expansion in patients with amyotrophic lateral sclerosis and frontotemporal dementia: a cross-sectional study. Lancet Neurol.

[2] Laaksovirta H, Launes J, Jansson L, Traynor BJ, Kaivola K, Tienari PJ (2022). ALS in Finland: major genetic variants and clinical characteristics of patients with and without the C9orf72 hexanucleotide repeat expansion. Neurol Genetics.

[3] Kaivola K, Salmi SJ, Jansson L, Launes J, Hokkanen L, Niemi AK, Majamaa K, Lahti J, Eriksson JG, Strandberg T, Laaksovirta H (2020). Tienari PJ carriership of two copies of C9orf72 hexanucleotide repeat intermediate-length alleles is a risk factor for ALS in the finnish population. Acta Neuropathol Commun.

[4] de Boer SCM, Woolley L, Mol MO, Serpente M, Reus LM, van Minkelen R, van Vugt JFA, Sorrentino F, Veldink JH, Seelaar H, Galimberti D, van Ruissen F, Mead S, Rogaeva E, Pijnenburg YAL, van der Lee SJ. Letter to the editor on a paper by Kaivola et al (2020): carriership of two copies of C9orf72 hexanucleotide repeat intermediate-length alleles is not associated with amyotrophic lateral sclerosis or frontotemporal dementia. Acta Neuropathol Commun 10(1):141.10.1186/s40478-022-01438-0 10.1186/s40478-022-01438-0 PMC9494883 36131298

[5] van der Zee J, Gijselinck I, Dillen L, Van Langenhove T, Theuns J, Engelborghs S, Philtjens S, Vandenbulcke M, Sleegers K, Sieben A, Baumer V, Maes G, Corsmit E, Borroni B, Padovani A, Archetti S, Perneczky R, Diehl-Schmid J, de Mendonca A, Miltenberger-Miltenyi G, Pereira S, Pimentel J, Nacmias B, Bagnoli S, Sorbi S, Graff C, Chiang HH, Westerlund M, Sanchez-Valle R, Llado A, Gelpi E, Santana I, Almeida MR, Santiago B, Frisoni G, Zanetti O, Bonvicini C, Synofzik M, Maetzler W, Vom Hagen JM, Schols L, Heneka MT, Jessen F, Matej R, Parobkova E, Kovacs GG, Strobel T, Sarafov S, Tournev I, Jordanova A, Danek A, Arzberger T, Fabrizi GM, Testi S, Salmon E, Santens P, Martin JJ, Cras P, Vandenberghe R, De Deyn PP, Cruts M, Van Broeckhoven C, van der Zee J, Gijselinck I, Dillen L, Van Langenhove T, Theuns J, Philtjens S, Sleegers K, Baumer V, Maes G, Corsmit E, Cruts M, Van Broeckhoven C, van der Zee J, Gijselinck I, Dillen L, Van Langenhove T, Philtjens S, Theuns J, Sleegers K, Baumer V, Maes G, Cruts M, Van Broeckhoven C, Engelborghs S, De Deyn PP, Cras P, Engelborghs S, De Deyn PP, Vandenbulcke M, Vandenbulcke M, Borroni B, Padovani A, Archetti S, Perneczky R, Diehl-Schmid J, Synofzik M, Maetzler W, Muller Vom Hagen J, Schols L, Synofzik M, Maetzler W, Muller Vom Hagen J, Schols L, Heneka MT, Jessen F, Ramirez A, Kurzwelly D, Sachtleben C, Mairer W, de Mendonca A, Miltenberger-Miltenyi G, Pereira S, Firmo C, Pimentel J, Sanchez-Valle R, Llado A, Antonell A, Molinuevo J, Gelpi E, Graff C, Chiang HH, Westerlund M, Graff C, Kinhult Stahlbom A, Thonberg H, Nennesmo I, Borjesson-Hanson A, Nacmias B, Bagnoli S, Sorbi S, Bessi V, Piaceri I, Santana I, Santiago B, Santana I, Helena Ribeiro M, Rosario Almeida M, Oliveira C, Massano J, Garret C, Pires P, Frisoni G, Zanetti O, Bonvicini C, Sarafov S, Tournev I, Jordanova A, Tournev I, Kovacs GG, Strobel T, Heneka MT, Jessen F, Ramirez A, Kurzwelly D, Sachtleben C, Mairer W, Jessen F, Matej R, Parobkova E, Danel A, Arzberger T, Maria Fabrizi G, Testi S, Ferrari S, Cavallaro T, Salmon E, Santens P, Cras P, European Early-Onset Dementia Consortium (2013). A pan-European study of the C9orf72 repeat associated with FTLD: geographic prevalence, genomic instability, and intermediate repeats. Hum Mutat.

[6] Gijselinck I, Van Mossevelde S, van der Zee J, Sieben A, Engelborghs S, De Bleecker J, Ivanoiu A, Deryck O, Edbauer D, Zhang M, Heeman B, Baumer V, Van den Broeck M, Mattheijssens M, Peeters K, Rogaeva E, De Jonghe P, Cras P, Martin JJ, de Deyn PP, Cruts M, Van Broeckhoven C (2016). The C9orf72 repeat size correlates with onset age of disease, DNA methylation and transcriptional downregulation of the promoter. Mol Psychiatry.

[7] Beck J, Poulter M, Hensman D, Rohrer JD, Mahoney CJ, Adamson G, Campbell T, Uphill J, Borg A, Fratta P, Orrell RW, Malaspina A, Rowe J, Brown J, Hodges J, Sidle K, Polke JM, Houlden H, Schott JM, Fox NC, Rossor MN, Tabrizi SJ, Isaacs AM, Hardy J, Warren JD, Collinge J, Mead S (2013). Large C9orf72 hexanucleotide repeat expansions are seen in multiple neurodegenerative syndromes and are more frequent than expected in the UK population. Am J Hum Genet.

[8] Kaivola K, Kiviharju A, Jansson L, Rantalainen V, Eriksson JG, Strandberg TE, Laaksovirta H, Renton AE, Traynor BJ, Myllykangas L, Tienari PJ (2019) C9orf72 hexanucleotide repeat length in older population: normal variation and effects on cognition. Neurobiol Aging 84:242e7–242e12. 10.1016/j.neurobiolaging.2019.02.026 10.1016/j.neurobiolaging.2019.02.026 PMC8981799 30979436

[9] Thys RG, Wang YH (2015). DNA replication dynamics of the GGGGCC repeat of the C9orf72 gene. J Biol Chem.

[10] Rostalski H, Korhonen V, Kuulasmaa T, Solje E, Krüger J, Gen F, Kaivola K, Eide PK, Lambert JC, Julkunen V, Tienari PJ, Remes AM, Leinonen V, Hiltunen M, Haapasalo A (2021). A novel genetic marker for the C9orf72 repeat expansion in the finnish population. J Alzheimers Dis.

